# Construction and immunogenicity of a DNA vaccine coexpressing GP3 and GP5 of genotype-I porcine reproductive and respiratory syndrome virus

**DOI:** 10.1186/1746-6148-10-128

**Published:** 2014-06-10

**Authors:** Jing-Qiang Ren, Wen-Chao Sun, Hui-Jun Lu, Shu-Bo Wen, Jie Jing, Fu-Long Yan, Hao Liu, Cun-Xia Liu, Peng-Peng Xiao, Xing Chen, Shou-Wen Du, Rui Du, Ning-Yi Jin

**Affiliations:** 1 Institute of Military Veterinary, Key Laboratory of Jilin Province for Zoonosis Prevention and Control, Academy of Military Medical Sciences, Changchun 130122, China; 2 College of Veterinary Medicine, Jilin University, Changchun 130062, China; 3 College of Animal Science and Technology, Graduate School, Jilin Agricultural University, Changchun 130118, China; 4 Institute of Special Animal and Plant Sciences, Chinese Academy of Agricultural Sciences, Changchun 130122, China

**Keywords:** Genotype-I PRRSV, GP3, GP5, DNA vaccine, Immunogenicity, Quil A

## Abstract

**Background:**

The European (EU) genotype of porcine reproductive and respiratory syndrome virus (Genotype-I PRRSV) has recently emerged in China. The coexistence of Genotype-I and -II PRRSV strains could cause seriously affect PRRSV diagnosis and management. Current vaccines are not able to protect against PRRSV infection completely and have inherent drawbacks. Thus, genetically engineered vaccines, including DNA vaccine and live vector engineered vaccines, have been developed. This study aimed to determine the enhanced immune responses of mice inoculated with a DNA vaccine coexpressing GP3 and GP5 of a Genotype-I PRRSV.

**Results:**

To evaluate the immunogenicity of GP3 and GP5 proteins from European-type PRRSV, three DNA vaccines, pVAX1-EU-ORF3-ORF5, pVAX1-EU-ORF3 and pVAX1-EU-ORF5, were constructed, which were based on a Genotype-I LV strain (GenBank ID: M96262). BALB/c mice were immunized with the DNA vaccines; delivered in the form of chitosan-DNA nanoparticles. To increase the efficiency of the vaccine, Quil A (Quillaja) was used as an adjuvant. GP3 and GP5-specific antibodies, neutralizing antibodies and cytokines (IL-2, IL-4, IL-10 and IFN gamma) from the immunized mice sera, and other immune parameters, were examined, including T-cell proliferation responses and subgroups of spleen T-lymphocytes. The results showed that ORF3 and ORF5 proteins of Genotype-I PRRSV induced GP3 and GP5-specific antibodies that could neutralize the virus. The levels of Cytokines IL-2, IL-4, IL-10, and IFN–γ of the experimental groups were significantly higher than those of control groups after booster vaccination (P < 0.05). The production of CD3^+^CD4^+^ and CD3^+^CD8^+^ T lymphocyte was also induced. T lymphocyte proliferation assays showed that the PRRSV LV strain virus could stimulate the proliferation of T lymphocytes in mice in the experimental group.

**Conclusions:**

Using Quil A as adjuvant, Genotype-I PRRSV GP3 and GP5 proteins produced good immunogenicity and reactivity. More importantly, better PRRSV-specific neutralizing antibody titers and cell-mediated immune responses were observed in mice immunized with the DNA vaccine co-expressing GP3 and GP5 proteins than in mice immunized with a DNA vaccine expressing either protein singly. The results of this study demonstrated that co-immunization with GP3 and GP5 produced a better immune response in mice.

## Background

Porcine reproductive and respiratory syndrome (also known as “blue ear disease”) is a highly contagious disease of pigs caused by porcine reproductive and respiratory syndrome virus (PRRSV). The disease causes reproductive failure in pregnant sows; in particular it causes a respiratory disease characterized by reproductive failure (late miscarriage, stillbirth, mummified, weak or tired piglets) in pregnant sows [1]. According to its antigenic differences, PRRSV can be divided into two subgroups: The European-type subgroup A (Genotype-I) reported by Wensvoort in 1991; the European representative strain is the LV strain [2]; and the North American-type Subgroup B (Genotype-II) reported by Benfield in 1992; the U.S. representative strain is VR-2332 [3]. The nucleotide sequence similarity of the two subgroups ranges from 54% to 67%. In the past, the predominant strains in Europe were subgroup A, while in the United States and the Asia-Pacific region, B subgroups predominated. Genetic analysis of Chinese PRRSV isolates showed that the main subgroup is the North American type. Recently, however, PRRSV has broken its geographical constraints. Genotype-I PRRSV has been reported in Asia and North America, and American wild-type PRRSV has been isolated in Europe [4,5]. The coexistence of Genotype-I and -II PRRSV strains could cause problems for PRRSV diagnosis and management. In recent years, several European type PRRSV field isolates were reported in Asian countries, such as South Korea and Thailand. Genotype-I PRRSV was also reported in China. Various research groups have isolated Genotype-I PRRSV in China [6-8]. Importantly, the appearance of European type PRRSV in China presented a significant challenge to the prevention and control of PRRSV, increasing the difficulty of analyzing the highly pathogenic PRRSV and the molecular mechanisms of immunization.

PRRSV has a single-stranded, positive strand, non-segmented RNA genome of about 15.0 kb. It contains nine open reading frames (ORFs), and adjacent ORFs partially overlap. ORF3 of PRRSV encodes the GP3 protein, which shows approximately 54% to 60% amino acid identity between North American and European isolates. In addition, GP3 can accommodate cysteine mutations and influence the reproductive capacity of PRRSV [9]. Experiments using a specific monoclonal antibody against the GP3 protein of the LV strain suggested that the GP3 protein is inserted into the virus particle or is a virus envelope-associated protein [10]. The GP3 protein plays an important role in viral infectivity and may induce cellular immunity. GP5 is a glycosylated protein, known as the E protein. GP5 has six epitopes that can induce specific neutralizing antibodies. The neutralizing ability of the antibodies is stronger than those induced by GP4 [2,9,11].

Pigs infected with PRRSV generate a series of anti-PRRSV specific antibodies; however, these antibodies cannot completely remove PRRSV and the immune response is slow. PRRSV interferes with induction of the cellular innate immune response, which is closely linked with apoptosis. These may explain the slow porcine immune response; however, the mechanism of this interference remains unclear [11]. Viral epitopes that could induce neutralizing antibodies are located in the M, GP3 and GP5 proteins. Epitopes that can induce antibody-dependent enhancement (ADE)-mediated effects are located in protein N and GP5 [12]. Currently, there are two types of commercial vaccines for PRRSV: modified live-attenuated vaccines (MLVs) and killed vaccines. However, both of them have inherent drawbacks. Killed vaccines are weakly immunogenic and cannot always provide protective immunity against PRRSV infection [13]. Although MLVs can provide a certain degree of protection against PRRSV, there is a possibility that the attenuated virus could return to high virulence [14]. Thus, there is an urgent need to develop more effective vaccines against PRRSV. DNA vaccines are a new generation of safe vaccines, and immunization with DNA vaccines elicit both cell-mediated and humoral immune responses [15-17].

Vaccines require optimal adjuvants, including immunopotentiator and delivery systems, to offer long term protection from infectious diseases in animals. Chitosan has well-defined properties including bioavailability, biocompatibility, low cost and an ability to open intracellular tight junction [18,19]. Therefore, chitosan, combined with advances in nanotechnology, can be effectively applied as a carrier system for vaccine delivery. Quillaja (Quil A) is a promising adjuvant that has been used in numerous prophylactic and therapeutic vaccines. Quil A modulates the cell mediated immune system as well as enhancing antibody production. In addition, only a low dose is needed for its adjuvant activity [20,21]. In this study, DNA vaccines pVAX1-EU-ORF3-ORF5, pVAX1-EU-ORF3 and pVAX1-EU-ORF5 were constructed based on the European LV strain (M96262) and formulated together with chitosan. Quil A was used as an adjuvant to immunize mice with individual DNA vaccines and their immunogenicities were evaluated using animal experiments.

## Results

### Purification of recombinant proteins GP3/GP5

**Figure 1:**
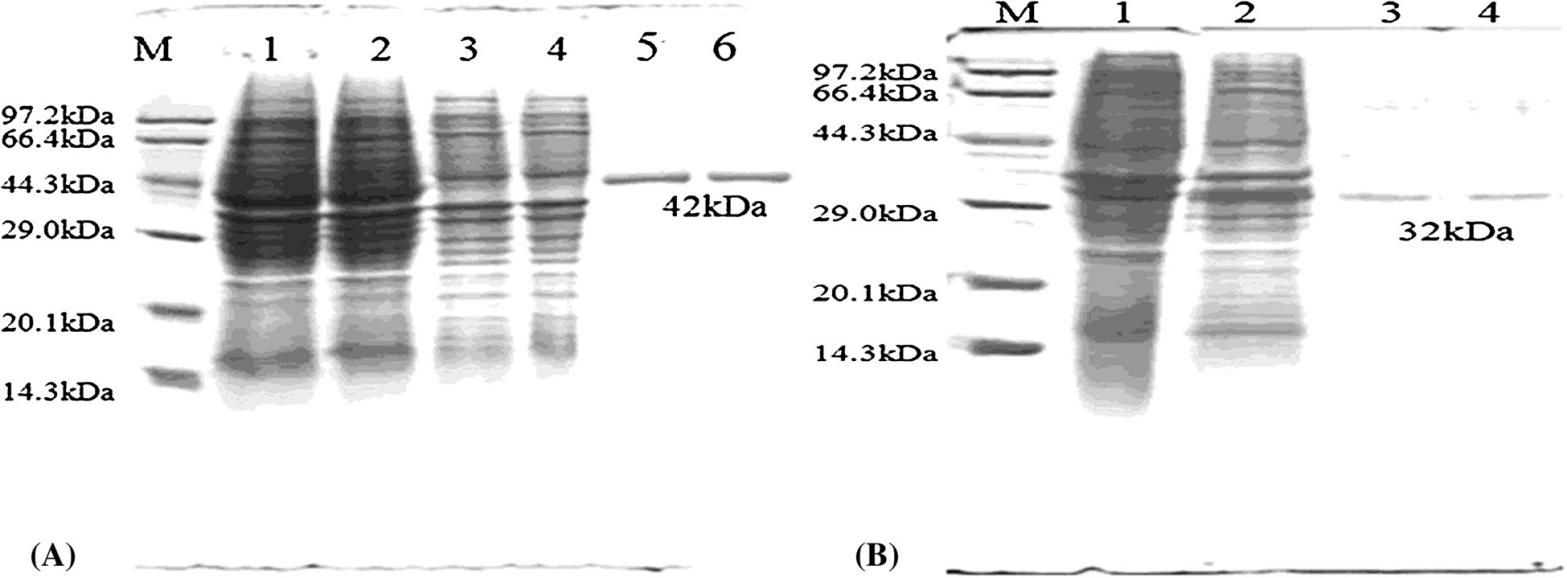
**European type PRRSV GP5/GP3 prokaryotic protein expression and purification. (A)** Protein expressed from pGEX-4 T-ORF5: M, molecular weight markers; lane 1 and 2, GP5 protein after IPTG induction; lane 3 and 4, empty vector control before IPTG induction; lane 5 and 6, purified protein. **(B)** Protein expressed from pET-28a-ORF3: M, molecular weight markers; lane 1 and 2, GP3 protein after IPTG induction; lane 3 and 4, purified protein.

The recombinant proteins expressed from pET-28a-ORF3 and pGEX-4 T-ORF5 were recovered separately and subjected to SDS-PAGE (Figure 1). pGEX-4 T-ORF5 expressed a protein of 42 kDa and pET-28a-ORF3 expressed a protein of 35 kDa, both of the expected size.

### Identification of expression GP3 and GP5 from the DNA vaccines

**Figure 2:**
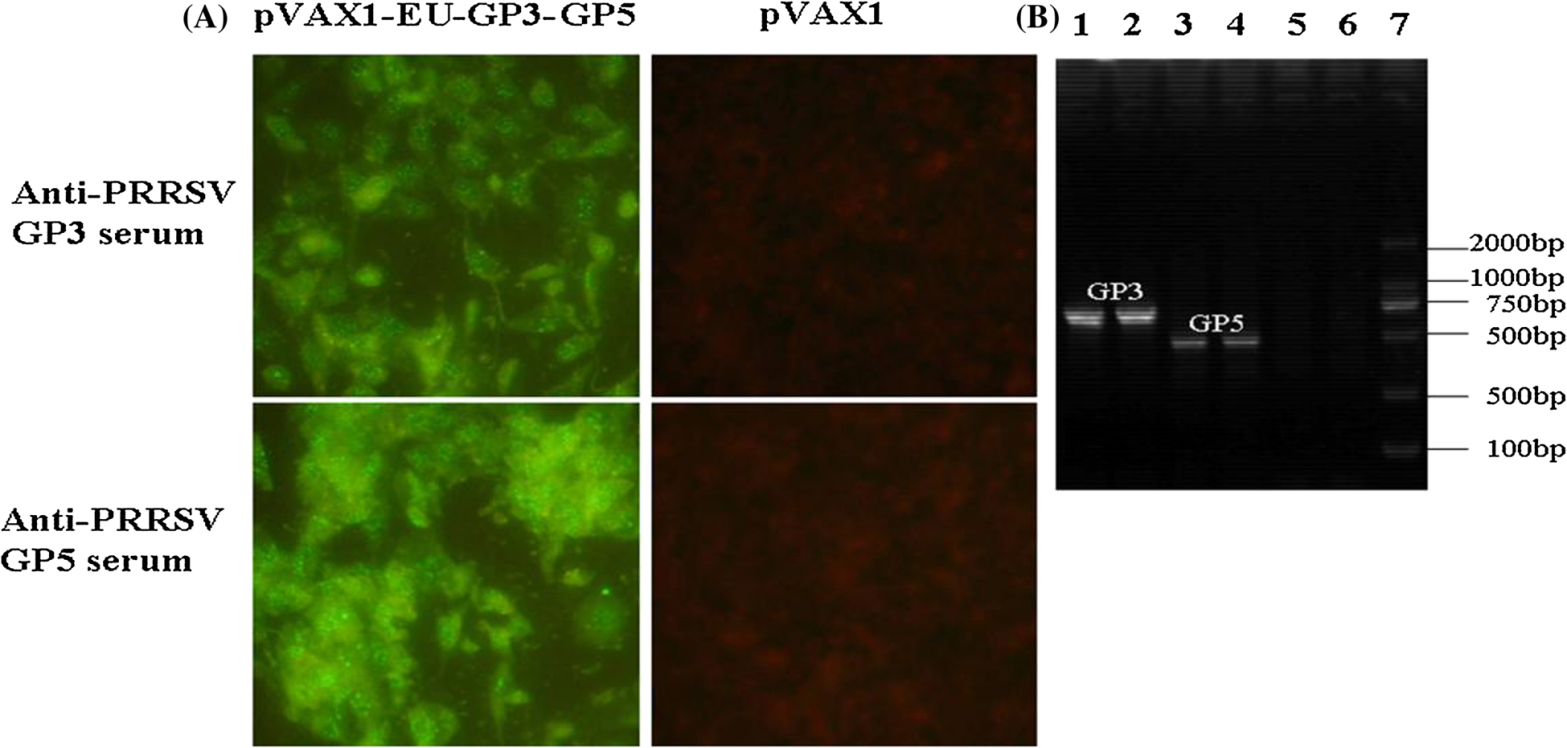
**Identification of expression of foreign proteins in vitro.** The expression of GP3 and GP5 of PRRSV were confirmed by an indirect immunofluorescence assay (IFA) following incubation with anti-PRRSV GP3 or GP5 protein antibody in BHK-21 cells **(A)**. RT-PCR was performed to detect expression of the GP3 and GP5 mRNAs **(B)**.

To determine whether the viral proteins were expressed from the DNA vaccines or not, BHK-21 cells were transfected with recombinant DNA vaccines pVAX1-EU-GP3-GP5 and recombinant vaccinia viruses rddVTT-GP3-GP5 and their protein amd mRNA expressions were detected by an indirect immunofluorescence assay (IFA) and RT-PCR (Figure 2). The IFA showed that recombinant vaccines transfected or infected into BHK-21 cells could be labeled with PRRSV-specific antibodies (Figure 2A), but cells transfected with pVAX1 control were not labeled, proving that GP3 and GP5 were expressed in vitro. After transfection (72 hours), the GP3 and GP5 mRNA could be detected by RT-PCR (Figure 2B).

### Detection of specific antibodies against GP3 and GP5 in immune sera

**Figure 3:**
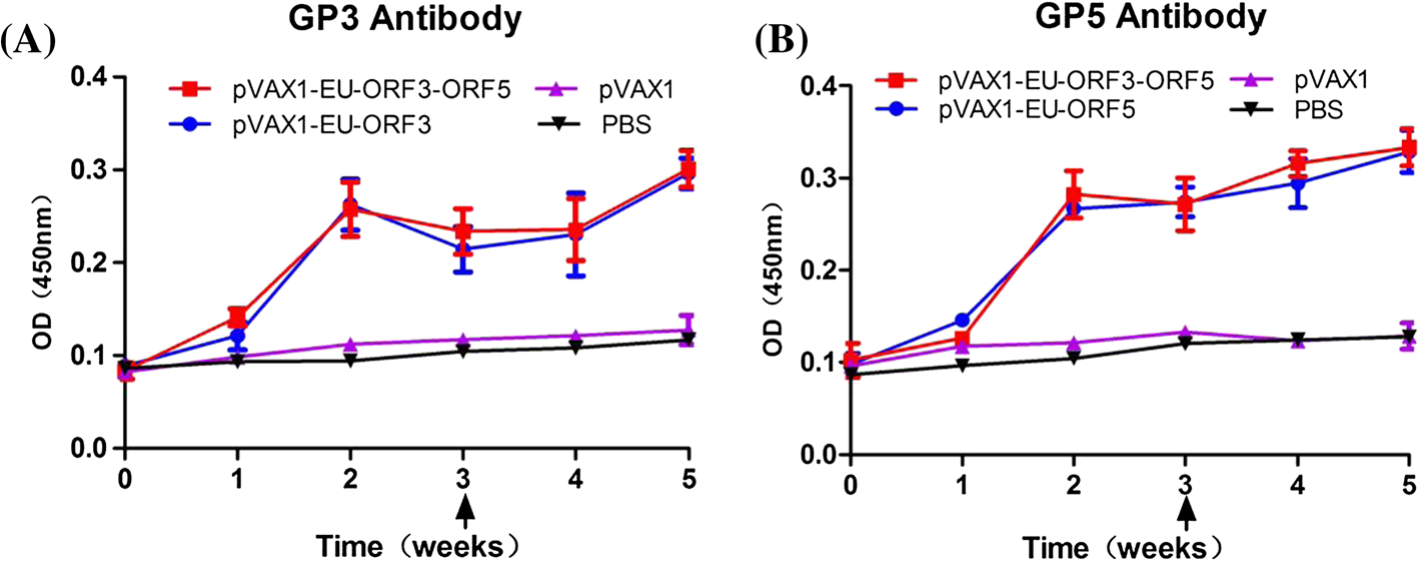
**ELISA assay for GP3 (A) or GP5 (B) specific antibodies in sera from mice immunized with recombinant DNA vaccines.** The antibody levels in mice immunized with DNA vaccines were compared with control mice immunized with pVAX1 empty vector or PBS. Serum samples (n = 6) were collected at various time-points. Arrows indicate the time of administration of boost immunizations. Data are shown as the mean ± S.D.

In the sera of mice immunized the DNA vaccines expressing GP3 and GP5, specific antibodies could be detected one week after immunization. However, compared with the control group at 7 dpi, the difference was not significant (P > 0.05). The antibody levels continued to rise and showed a statistically significant difference compared with the control group after two weeks (P < 0.05). A slight decrease was observed in the third week. After the booster immunization at 21 days, the antibody levels increased to a peak at 35 days post immunization (dpi). The antibody levels in the pVAX1-EU-ORF3-ORF5 group were slightly higher than in the other two experimental groups; however, the difference was not statistically significant (Figure 3).

### Detection of neutralizing antibodies in sera

Sera from immunized mice were collected and separated weekly to detect PRRSV neutralizing antibody titers from two weeks after immunization. The neutralizing antibody titer in mice immunized with the DNA vaccine co-expressing GP3 and GP5 protein (pVAX1-EU-ORF3-ORF5) was significantly higher (P < 0.05) than that in the groups immunized with pVAX1-EU-ORF5 and pVAX1-EU-ORF3 (P < 0.01), and peaked at 42 dpi (1:21.1) (Table 1). The neutralizing antibody titers of each experimental group were significantly higher than control groups (pVAX1, PBS) (P < 0.05) at 42 dpi; however, the neutralizing antibodies produced by the pVAX1-EU-ORF3 group had only a weak ability to neutralize the virus.

**Table 1:** Neutralizing antibody titers of PRRSV LV strain in mice immunized with different DNA vaccines

**Immunized groups**	**7 dpi**	**14 dpi**	**28 dpi**	**42 dpi**
pVAX1-EU-ORF3	<2	4.6 ± 0.22	7.3 ± 0.68	6.8 ± 0.23
pVAX1-EU-ORF5	<2	6.4 ± 0.32	10.9 ± 0.97	16.3 ± 1.45
pVAX1-EU-ORF3-ORF5	<2	8.5 ± 0.65	14.8 ± 1.28	21.1 ± 2.03
pVAX1	<2	<2	<2	<2
PBS	<2	<2	<2	<2

Virus-neutralizing (VN) antibody titers in mice after vaccination for different groups. Serum samples (n = 8) were collected at various time-points. PRRSV-specific neutralizing antibodies were detected by a virus neutralizing assay with two-fold serial dilutions. The VN titers were expressed as the reciprocal of the highest serum dilution in which no CPE was observed.

### Levels of secreted cytokines IL-2 and IFN-γ after immunization

**Figure 4:**
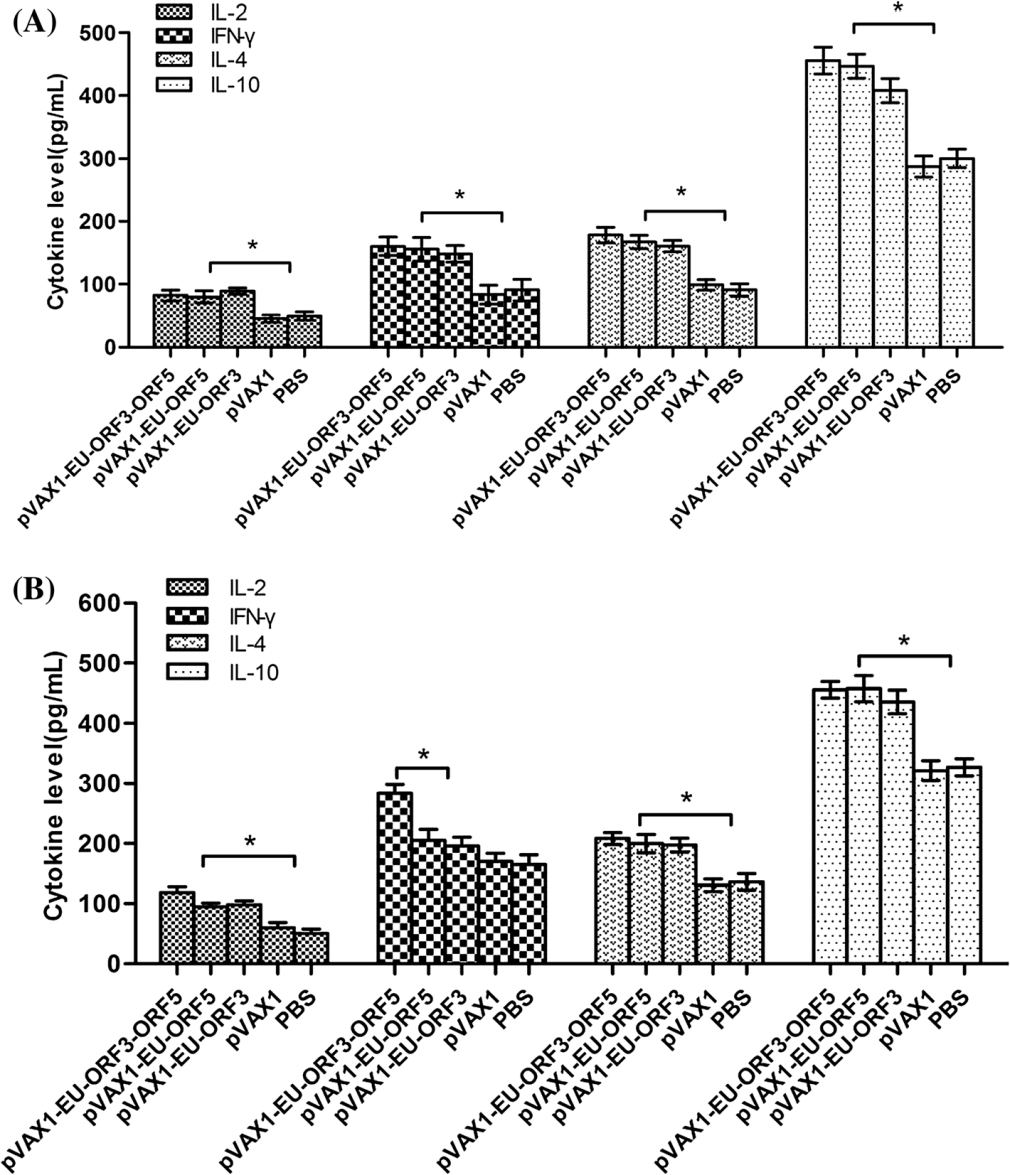
**Detection of IL-2, IL-4, IL-10 and IFN-γ secretion levels in immune serum. (A)** Cytokine secretion levels in serum at 14 days post immunization (dpi); **(B)** cytokine secretion levels in serum at 35 dpi. * Indicates a significant (P < 0.05) difference between the groups. Data are shown as the mean ± S.D.

The levels of cytokines IL-2 and IFN-γ, the main representative Th1 cytokines, were detected in serum separated from collected blood at 14 dpi and 35 dpi. The levels of IL-2 in the experimental groups (pVAX1-EU-ORF3, pVAX1-EU-ORF5, pVAX1-EU-ORF3-ORF5) were significantly higher than in the control groups (pVAX1, PBS) (P < 0.01) at 14 and 35 dpi; however, no significant difference was observed among the experimental groups (P > 0.05). At 35 dpi, the levels of IFN-γ in the pVAX1-EU-ORF3-ORF5 group were significantly higher than those in any of the other groups (P < 0.05) (Figure 4A and B). These results not only demonstrated that the constructed DNA vaccines could effectively stimulate mice to produce specific Th1 lymphocytes, contributing to the secretion of IL-2 and IFN-γ (and possibly other Th1 cytokines), but also suggested the DNA vaccine could induce a cellular immune response in mice. In addition, the combined antigen group showed a synergistically enhanced immune reaction in terms of secretion of IFN-γ and was superior to either single-antigen DNA vaccine.

### The secretion levels of cytokines IL-4 and IL-10 after immunization

The levels of cytokines IL-4 and IL-10, the main representative of Th2 cytokines, were detected in sera at 14 dpi and 35 dpi. The IL-4 and IL-10 levels in the experimental group were significantly higher (P < 0.05) than those in the control groups (pVAX1, PBS) at 14 and 35 dpi; however, no significant difference was observed among the experimental groups (P > 0.05). At 35 dpi, the levels of IL-4 and IL-10 in the pVAX1-EU-ORF3-ORF5 group were slightly, but not statistically significantly, higher than those in the pVAX1-EU-ORF3 group (Figure 4A and B). These results indicated that the DNA vaccine could effectively stimulate the body to increase the secretion of Th2 cytokines related to the humoral immune response.

### T lymphocyte proliferation response of mice

**Figure 5:**
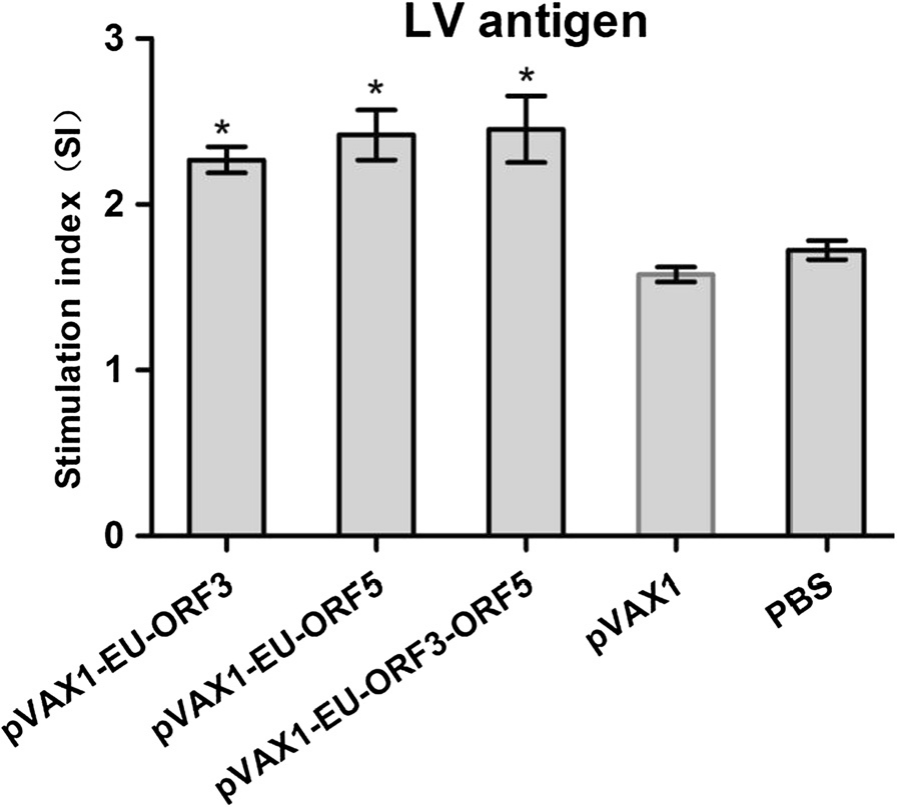
**Lymphocyte proliferative responses in immunized mice after in vitro stimulation with purified PRRSV LV antigen.** Each group of mice (n = 6) was immunized with 100 μg of DNA vaccine at 0 and 3 weeks. Five weeks after the last inoculation, the mice were sacrificed and their splenocytes stimulated with PRRSV virus. After 72 h of stimulation, WST-1 was added and OD values were determined after a further 4 h of incubation. The samples were assayed in triplicate. Data are presented as the mean ± standard error. * Indicates a significant difference (P < 0.05) between the groups. Data are shown as the mean ± S.D.

Immunized mice stimulated with the non-specific antigen ConA showed T lymphocyte proliferation, and the stimulation index (SI) was significantly higher than the control groups (pVAX1, PBS) (P < 0.05; data not shown). Upon stimulation with the PRRSV LV strain virus antigen, the experimental group produced a specific T lymphocyte proliferative response, and the SI difference was significant compared with the control group (P < 0.05); however, the differences among the experimental groups were not significant (Figure 5).

### FACS analysis of T lymphocyte subgroups from the spleens of immunized mice

**Figure 6:**
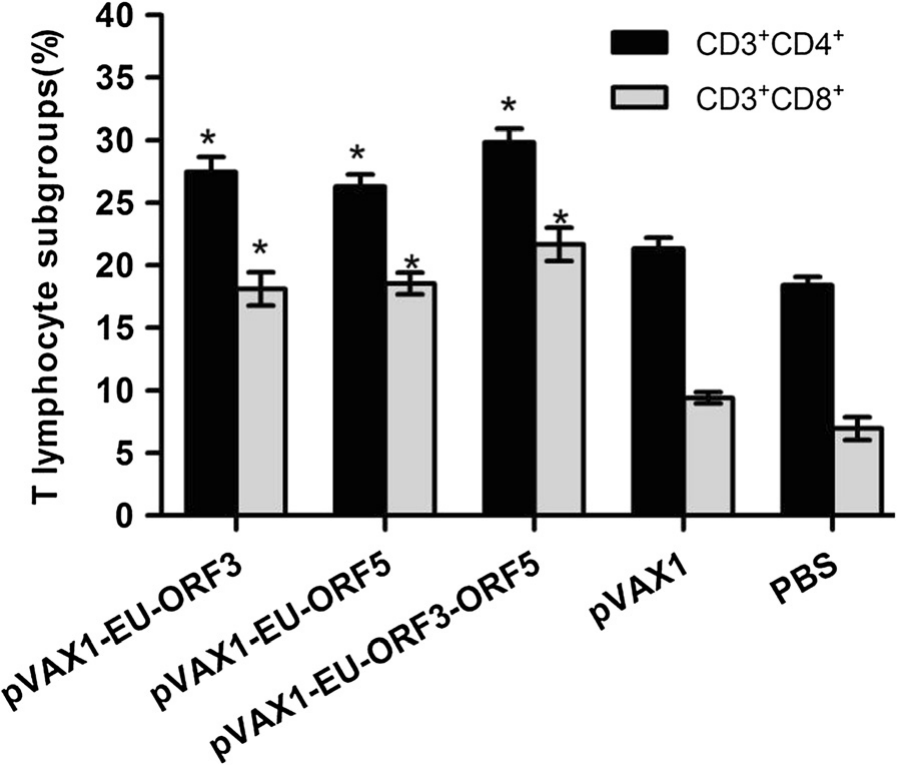
**Detection of T cell subgroups of spleens in mice.** Splenocytes of mice (n = 8) were harvested and stained as described in Methods section. FACS was used to analyze the percentage of CD3^+^CD4^+^ or CD3^+^CD8^+^ T cells. Each bar represents the group mean for the percentage of T cell subgroups. * Indicates a significant difference (P < 0.05) between the groups. Data are shown as the mean ± S.D.

Two weeks after the second immunization, splenic lymphocytes were isolated and analyzed for CD3^+^CD4^+^ and CD3^+^CD8^+^ T lymphocytes. As shown in Figure 6, the percentages of CD3^+^CD4^+^ and CD3^+^CD8^+^ T cells in each experimental group were significantly higher than those in the control groups (pVAX1, PBS) (P < 0.05). Mice inoculated with pVAX1-EU-ORF3-ORF5 showed slightly higher levels of CD3^+^CD4^+^ and CD3^+^CD8^+^ T cells than mice inoculated each single antigen. This indicated that DNA vaccines coexpressing ORF3 and ORF5 gene could stimulate murine CD4^+^ and CD8^+^ T lymphocyte proliferation, and induce both humoral and cellular immune responses.

## Discussion

PRRSV epidemics have resulted in the virus being spread throughout the world, causing huge economic losses to the pig industry. In the past, European strains of PRRSV were limited to European countries, but have now spread to Asia and North America [22]. South Korea, Thailand, China and other countries have reported many European type PRRSV strains in recent years [23,24]. European strains Ningbo42 (EF473137) and FJ0603 (EF592535) were isolated and found to be closely related to the Genotype-I PRRSV attenuated vaccine strain AMERVAC-PRRS/A3 [25]. The FJ0602 strain (HM755885) has also been proven to be non-pathogenic to nursery pigs [26]. Two strains of wild-type Genotype-I PRRSV, BJEU06-1 (GU047344) and NMEU09-1 (GU047345), were isolated from a pig compound in 2011 [27], which was the first report of Genotype-I PRRSV field strains in China. The appearance of Genotype-I PRRSV in China complicates the prevention and control of PRRSV. Genotype-I and -II PRRSV vaccines cannot provide cross-protection, and these two strains may produce recombinant virus, chimeric virus or a new virus, while the selection pressure exerted by antibodies could significantly affect the genetic variation of PRRSV and change its antigenicity [28,29].

Vaccination is the most effective and valuable tool to prevent infectious diseases. In this study, DNA vaccines based on Genotype-I PRRSV were constructed. The DNA vaccines were formulated together with chitosan and delivered in the form of chitosan-DNA nanoparticles. Chitosan has wide applications in biotechnology, pharmaceuticals, textiles, food, cosmetics and agricultural industries [30]. Research has focused on chitosan’s use as a novel delivery vehicle for drugs, genes, peptides and vaccines, and as a scaffold for targeted delivery and tissue engineering applications [31-33]. Chitosan effectively binds DNA via electrostatic interactions and protects it from nuclease degradation [34], which enhances transfection efficiency both *in vitro* and *in vivo*[35]. To increase the efficiency of the vaccine, Quil A (Quillaja) was used as an adjuvant when immunizing mice with individual DNA constructs. One week after immunization, specific antibodies to GP3 and GP5 could be detected. Three weeks after the booster immunization, the antibody levels continued to increase and were significantly higher than in the control groups. Neutralizing antibodies were detected two weeks after immunization (usually they can only be detected after three weeks), probably because Quil A enhanced the immune effect of the DNA vaccine. Quil A (Quillaja) is extracted from the evergreen tree Quillaja saponaria as triterpenoid compounds [36], which activate Th cells, cytotoxic T lymphocytes and B-cells. Quil A improves the immune reaction of an antibody to an antigen; improves the production of antibody subclasses IgG3, IgG2a and IgG2b; and enhances the secretion of IL-2, TNF-β and IFN-γ [37,38]. Neutralizing antibodies play an important role in the anti-PRRSV response. During PRRSV infection, the induction of neutralizing antibodies indicates that the virus has begun to be cleared from the tissues and blood. Previous studies showed that the GP3 protein of European strains has a neutralizing epitope between amino acids 57 and 73 [39]; however, the detailed protein structure and function require further study. The data presented here showed that GP3 and GP5 could induce neutralizing antibodies in mice; however, the GP3 neutralizing antibody titer was low. Co-expression of GP3 and GP5 produced a synergistic effect, resulting in a better neutralizing antibody response. The GP5 protein could induce specific neutralizing antibodies and serotype-specific linear epitopes could neutralize viral infections in vitro. A previous study showed that the neutralizing ability of GP5 was higher than that of GP4 and virus neutralization was significantly correlated with GP5 antibody titers [40].

In viral diseases, removal of the virus via cellular immunity plays an important role in the prevention of disease. Cell-mediated immunity (CMI) is also extremely important in PRRSV infection [41]. Previous studies have shown that CMI is significantly related to reduced clinical symptoms in PRRSV-infected pigs [42]. The PRRSV-specific CMI response appears approximately 2–4 weeks after vaccination, as determined by lymphocyte proliferation and interferon γ (IFN-γ) production in a recall reaction [43,44].

To detect the T cell-mediated immune response, we isolated mouse spleen lymphocytes and performed lymphocyte proliferation transformation experiments in vitro. We found that the experimental group could induce specific T cell proliferative responses after stimulation by a PRRSV LV strain virus-specific antigen. These results also indicated that, in each experimental group, the levels of CD4^+^ and CD8^+^ T cells were significantly higher (P <0.05) than those in the PBS and pVAX1 immunized group (P <0.01). In the pVAX1-EU-ORF3-ORF5 immunized group, the levels of CD4^+^ and CD8^+^ were higher than those in groups immunized with the single protein DNA vaccines. The percentage of CD4^+^ T cells in the circulating peripheral blood is directly related to the severity of the disease in an animal. The smaller the proportion of CD4^+^ T cells, the more likely that the animals will develop a serious infection. CD4^+^CD8^+^ immune cells have an important function in antigen recognition. The immune response mediated by Th1 cells could be affected by CD8^+^ T cells in PRRSV infection [45,46]. Th1 cells in CD4^+^ subsets (Th cells) secrete IL-2, IFN-γ, TNF-β and other cytokines. Th1 cells mainly mediate the immune response and play important roles in immune regulation of organ-specific autoimmune diseases, in organ transplant rejection and infection immunity. Th2 cells in CD4^+^ subsets (Th cells) secrete IL-4, IL-5, IL-6, IL-10, IL-13 and other cytokines. Th2 cells mainly regulate humoral immune responses and play a decisive role in the induction of anaphylaxis. In this study, the assessment of serum cytokines after vaccination revealed that the pVAX1-EU-ORF3-ORF5 groups secreted significantly higher levels of IFN-γ than any other group at 35 dpi. While levels of IL-2 and IFN-γ peaked at 35 dpi, those of IL-4 and IL-10 reached a maximum at 35 dpi in the pVAX1-EU-ORF3-ORF5 group. These results indicated that mice immunized with DNA vaccines were induced to produce both Th1-type and Th2-t cytokines after the booster vaccination.

## Conclusions

In summary, the DNA vaccines expressing GP3 and GP5 of Genotype-I PRRSV showed good immunogenicity, and the DNA vaccine expressing both GP3 and GP5 produced better results. These data provided a theoretical basis for future experimental studies. In addition, careful selection of adjuvants or delivery systems can enhance prime-boost regimen-elicited immune responses, and new vaccine adjuvants can potentiate immunogenicity and the protective effect of PRRSV vaccines. Consequently, it is essential that future PRRSV vaccines must be more potent, safe, effective, and provide better protection against PRRSV. Furthermore, the involvement of GP3 and GP5 proteins in viral proliferation and viral replication during infection-induced immune responses also requires further research.

## Methods

### Viruses and cells

The Dalian Entry-Exit Inspection And Quarantine Bureau provided the Lelystadstrain of PRRSV (LV, its nucleotide sequence homology with those wild strains in China was 91%-97% [27,47], which also is the obtainable strain for us). Virus propagation in MARC-145 cells was performed in MEM medium supplemented with 10% fetal bovine serum at 37°C with 5% CO2. The Reed-Muench method [48] was used to determine the virus titers (expressed as 50% tissue culture infective dose [TCID_50_] per milliliter) on Marc-145 cells.

### Obtaining the target genes and construction of plasmids

A Viral RNA Mini Kit (QIAgen Inc., Valencia, CA, USA) was used to extract the viral RNA, according to the manufacturer’s instructions. Reverse transcription using a PrimeScript® RT Master Mix kit (TaKaRa Biotechnology Co. Ltd., Dalian, China) was used for first-strand cDNA synthesis, which were stored at -80°C. PCR was used to amplify ORF3 and ORF5 of PRRSV European type. Two prokaryotic expression plasmids (pET-28a-ORF3 and pGEX-4 T-ORF5), and three eukaryotic expression plasmids (pVAX1-EU-ORF3-ORF5, pVAX1-EU-ORF3 and pVAX1-EU-ORF5) were constructed. The GP5 N-terminal signal peptide sequence affects its expression in prokaryotic expression plasmids; therefore, the DNA sequence encoding the GP5 signal peptide sequence was removed in this study. A sequence encoding a G4S flexible linker was inserted between the ORF3 and ORF5 genes in the eukaryotic expression plasmid pVAX1-EU-ORF3-ORF5. All the primers used in this study are shown in Table 2.

**Table 2:** Primer sequences for amplification of ORF3 and ORF5 genes from PRRSV strain LV

**Name**	**Sequence (5′ → 3′)**
P1	CGCGGATCCGCTCATCAGTGTGCACGCTTCCAT
P2	CCCAAGCTTTCGTGATGTACTGGGGAGTACCG
P3	CGGGATCCGGCAACGGCGACAGCTC
P4	CCCTCGAGGGCCTCCCATTGCTCAG
P5	CGCGGATCCAGATGTTCTCACAAATTGGGGCGTT
P6	CCCAAGCTTGGCCTCCCATTGCTCAGCCG
P7	CGCGGATCC*GGAGGCGGAGGCTCCGGAGGAGGAGGCTCCGGAGGCGGAGGGTCT*AGATGTTCTCACAAATTGGGGCGTT

Primers P1/P2 were for full-length ORF3; P3/P4 were for ORF5 lacking the signal peptide; primers P5/P6 were for full-length ORF5, primer P7 was for the full-length sequence of ORF5; the italics part encodes the G4S flexible Linker.

### Purification of prokaryotic expressed proteins and the preparation of polyclonal antibodies

GP3 and GP5 protein, as structure envelope protein of PRRSV, can hardly be expressed by prokaryotic expression system, in this study, we deleted the signal peptide sequence to obtain GP3 and GP5 protein so as to ensure the expression level. The expression plasmids correctly identified as pET-28a-ORF3 and pGEX-4 T-ORF5 were induced by IPTG, and the expressed proteins were recovered from the bacteria and analyzed by SDS-PAGE. Six 3-month-old male New Zealand white rabbits were immunized with purified GP5 or GP3 protein mixed with an equal volume of Freund’s complete adjuvant. Two weeks later, a booster immunization in Freund’s incomplete adjuvant was administered. A third immunization was performed three weeks later. One week after the third immunization, rabbit sera were analyzed by western blotting.

### Immunofluorescence assays

An IFA was used to determine the protein expressions of the European-based DNA vaccines pVAX1-EU-ORF3-ORF5, pVAX1-EU-ORF3 and pVAX1-EU-ORF5 in BHK-21 cells, as previously described [49].

### Animal grouping and immunization

The Research Ethics Committee of Jilin University reviewed and approved all the procedures for handling the mice used in this study. All animal experiemnts were conducted in accordance with the Chinese Laboratory Animal Administration Act 1988. All mice experiments were performed in an ethical and humane manner under veterinary supervision.

Sixty 6-week-old female BALB/c mice, weight 18–20 g (Experimental Animal Center, Academy of Military Medical Science of PLA, Beijing), were randomly divided into five groups (12 mice each): the experimental groups were pVAX1-EU-ORF3-ORF5, pVAX1-EU-ORF3 and pVAX1-EU-ORF5; the control groups were pVAX1 and PBS. All mice were maintained and bred in the experimental animal facilities of the Institute of Military Veterinary. Each mouse was injected intramuscularly (IM) with a dose of 100 μg of plasmid DNA, and a booster immunization with same dose was performed after three weeks. The plasmid DNAs were delivered in the form of chitosan-DNA nanoparticles. After completely dissolving 0.2 g chitosan (Sigma-Aldrich, St. Louis, MO, USA) in 200 mL of 1% acetic acid, sodium hydroxide solution was added to adjust the solution to pH 5.5. The chitosan solution was stored at 4°C after sterile filtration through a 0.45 μm membrane filter. Plasmid DNA (100 μg) dissolved in 100 μL of 20 mmol/L sodium sulfate solution with 200 μg Quil A (Accurate Chemical & Scientific Corporation, Westbury, NY, USA) as adjuvant, was mixed with an equal volume of chitosan solution. Following a 30-min incubation in a water bath at 55°C, the solution was rapid mixed by vortexing for 30s, and agarose gel electrophoresis was used to evaluate the uniformity of the coated nanoparticles.

Sera from mice in the experimental groups were separated at 0, 7, 14, 21, 28, 35 and 42 days for the detection of specific antibodies and cytokine analysis of peripheral blood. Mice were sacrificed 14 days after the second immunization, and spleen T-lymphocytes were separated and analyzed for T cell subsets (CD3^+^CD4^+^ and CD3^+^CD8^+^).

### Detection of specific antibodies for GP3, GP5

Purified GP3 and GP5 recombinant proteins were diluted to 2 μg/ml with phosphate buffer (0.01 M, pH7.4) as coating antigen for an indirect ELISA to detect the levels of specific antibodies in sera. The protocol followed a previously published method [50].

### Serum neutralization assays

Sera from all animals in each immunization group were heat-inactivated for 30 min at 56°C. Serial two-fold dilutions of test sera were incubated for 60 min at 37°C in the presence of 200 TCID_50_ of the LV strain in DMEM containing 2% FBS. The mixtures were added to 96-well microtiter plates (Costar, Corning, Tewksbury, MA, USA) containing confluent MARC-145 cells (2 × 10^5^ cells,) that had been seeded 48 h earlier. After incubation for 5 days at 37°C in a humidified atmosphere containing 5% CO_2_, the cells were examined for cytopathic effects (CPEs). Meanwhile, positive and negative controls, virus regression tests, serum toxicity controls and normal cell controls were performed. Finally, according to the Spearman-Karber method, the dilution of serum that contained a neutralizing antibody titer that could protect 50% of cell from the CPE was calculated.

### Cytokines secretion assay

An ELISA kit (ELISA Ready-SET-Go!®, eBioscience, San Diego, CA, USA) detected serum IL-2, IL-4, IL-10 and IFN-γ, according to the manufacturer’s instructions.

### Preparation of mice spleen lymphocytes

Centrifugation with Ficoll-Hypaque lymphocyte isolation solution (TBD Science, China) was used to isolate splenocytes of immunized mice, according to the manufacturer’s instructions.

### T lymphocyte proliferation assay

To assess the proliferative response of T lymphocytes against LV-specific antigens, lymphocytes in RPMI 1640 were adjusted to 2 × 10^6^ cells/mL and 50 μL of lymphocytes (1 × 10^5^ cells) were added to wells of a 96 well plate. The control wells included a non-specific stimulant (ConA, 5 μg/mL, Sigma), 50 μL/well; a specific stimulant (PRRSV LV virus: normally, LV strain does not infect murine cells; T lymphocyte proliferation response approach is not through virus infection but use the virus as antigen stimulation to lead transform and proliferation of sensitized lymphocyte. In addition, purified virus as antigen stimulation will be more close to the native conformation of virus protein) 50 μL/well (1MOI); and no stimulation control cells (RPMI-1640), 50 μL/well. Each sample included three repetition wells. The 96-well cell culture plate was incubated in a 5% CO_2_ incubator at 37°C for about 72 h, and then 10 μl (5 mg/mL) WST-1 (Beyotime Institute of Biotechnology, Haimen, China) solution was added to each well, before incubating for 3–5 h in a 5% CO_2_ incubator at 37°C. An ELISA microplate reader measured the absorbance at 450 nm. The proliferation of splenocytes was represented by the stimulation index (SI): SI = mean absorbance value at A450 of stimulated cells divided by the mean absorbance value at A450 of the negative control.

### CD4^+^ and CD8^+^ T-cell subtype assay

Mouse spleen lymphocytes (1 × 10^6^) were transferred into a 1.5 mL centrifuge tube. One milliliter of a fluorescent solution (100 mL 0.15 M PBS pH7.4, 2%NBS) was added, and the tube was centrifuged at 1500 rpm for 3 to 5 minutes. The supernatant was removed, and the pellet was resuspended in 300 μL of cell fluorescence solution. PE anti-mouse CD8, PE/Cy5 anti-mouse CD3, FITC anti-mouse CD4 (BioLegend, CA, USA) fluorescent antibodies were added and thoroughly mixed before being placed in the dark at 4°C for 30 minutes. After washing twice with fluorescent solution and centrifuging at 1500 rpm for 5 minutes, the supernatant was discarded. The cell pellet was resuspended in 500 μL of fluorescent preservation solution (0.15 M PBS pH 7.4, 2% Glucose, 1% Formaldehyde, 0.1%NaN3). Flow cytometry was then used to count CD3^+^CD4^+^ and CD3^+^CD8^+^ T lymphocytes among 10,000 cells. Statistical analysis of the percentage of CD3^+^CD4^+^ and CD3^+^CD8^+^ T lymphocytes was then performed.

### Statistical analysis

All data are presented as mean ± S.D. The differences in the level of humoral and cellular immune responses between different groups were determined by One-way repeated measurement ANOVA and Least significance difference (LSD). Differences were considered statistically significant when P < 0.05.

## Abbreviations

EU: European; PRRSV: Porcine reproductive and respiratory syndrome virus; NA: North American; ORF: Open reading frame; LV: Lelystad virus; TCID50: 50% tissue culture infective dose; SI: Stimulation index; SDS-PAGE: Sodium dodecyl sulfate-polyacrylamide gel electrophoresis; DPI: Days post inoculation; CPE: Cytopathic effect; VN: Virus neutralizing.

## Competing interests

The authors declare that they have no competing interests.

## Authors’ contributions

JQR performed most of the experimental work and drafted the manuscript. WCS, SBW and JJ participated in the analysis of humoral and cellular responses. FLY, HL, CXL, PPX, XC and SWD participated in the immunization of mice. NYJ and HJL revised the manuscript for important intellectual content and gave final approval of the version to be published. All authors read and approved the final manuscript.

## Authors’ information

Submitting author, Jing-Qiang Ren, E-mail: rjq207@163.com; Address: Institute of Military Veterinary, No. 666 west liuying Road, Jingyue District, Changchun City, Jilin Prov. China 130122.

## References

[B1] Bilodeau R Dea S Sauvageau RA Martineau GP Porcine reproductive and respiratory syndrome in Quebec Vet Rec 1991 129 102 103 1926720 10.1136/vr.129.5.102

[B2] Wensvoort G Terpstra C Pol JM ter Laak EA Bloemraad M de Kluyver EP Kragten C van Buiten L den Besten A Wagenaar F Mystery swine disease in The Netherlands: the isolation of Lelystad virus Vet Q 1991 13 3 121 130 10.1080/01652176.1991.9694296 1835211

[B3] Benfield DA Nelson E Collins JE Harris L Goyal SM Robison D Christianson WT Morrison RB Gorcyca D Chladek D Characterization of swine infertility and respiratory syndrome (SIRS) virus (isolate ATCC VR-2332) J Vet Diagn Invest 1992 4 2 127 133 10.1177/104063879200400202 1616976

[B4] Thanawongnuwech R Thacker B Halbur P Thacker EL Increased production of proinflammatory cytokines following infection with porcine reproductive and respiratory syndrome virus and mycoplasmahyop neumoniae Clin Diagn Lab Immunol 2004 11 5 901 908 15358650 10.1128/CDLI.11.5.901-908.2004 PMC515260

[B5] Psikal I Moutelikova R Kosinova E Mojzis M Smid B Nejedla E Indik S Rodak L Molecular identification and genotyping of porcine reproductive and respiratory syndrome virus (PRRSV) strains in the Czech and Slovak Republics Proceedings of the 3rd International Symposium of PRRSV and Aujeszky 1999 1 175 176

[B6] Sun YJ Sun YF Quarantine and diagnosis of porcine reproductive and respiratory syndrome Chinese Journal of Veterinary Medicine 1997 23 2 8 9

[B7] Zhang ZF Li XL Zhang Y Cha YX Fang WH Establishment of RT-PCR method for diagnosing European-type PRRSV and sequencing and analysis of the ORF7 gene Progress in Veterinary Medicine 2007 28 5 26 29

[B8] Huang MQ Che YL Chen SY Jing B Wei H Wang LB Chen SL Zhou LJ Zhuang XS Isolation of European type porcine reproductive andrespiratory syndrome virus FJ0602 strain (PRRSV-FJ0602) and sequence analysis of the ORF7 gene Chin J Vet Med 2008 30 3 174 178

[B9] Feng CY Liu YH Yan JH Gao GF An infectious clone of the highly pathogenic porcine reproductive and respiratory syndrome virus: Topology of glycoprotein 3 (GP3) addressing the intrachain disulfide bonds Chinese Sci Bull 2011 56 2785 2793 10.1007/s11434-011-4631-8

[B10] Meulenberg JJ Petersen-Den BA De Kluyver EP Moormann RJ Schaaper WM Wensvoort G Characterization of proteins encoded by ORFs 2 to 7 of Lelystad virus J Virol 1995 206 155 163 10.1016/S0042-6822(95)80030-1 PMC7130653 7831770

[B11] Miller LC Fox JM Apoptosis and porcine reproductive and respiratory syndrome virus Vet Immunol Immunopathol 2004 102 131 142 10.1016/j.vetimm.2004.09.004 15507300

[B12] Ansari IH Kwon B Osorio FA Pattnaik AK Influence of N-linked glycosylation of porcine reproductiveand respiratory syndrome virus GP5 on virus infectivity, antigenicity, and ability to induce neutralizing antibodies J Virol 2006 80 8 3994 4004 10.1128/JVI.80.8.3994-4004.2006 16571816 PMC1440468

[B13] Meng XJ Heterogeneity of porcine reproductive and respiratory syndrome virus: implications for current vaccine efficacy and future vaccine development Vet Microbiol 2000 74 309 329 10.1016/S0378-1135(00)00196-6 10831854 PMC7117501

[B14] Botner A Strandbygaard B Sorensen KJ Have P Madsen KG Madsen ES Alexandersen S Appearance of acute PRRS-like symptoms in sow herds after vaccination with a modified live PRRS vaccine Vet Rec 1997 141 497 499 10.1136/vr.141.19.497 9402722

[B15] Donnelly JJ Ulmer JB Liu MA DNA vaccines Life Sci 1997 60 163 172 9000640 10.1016/s0024-3205(96)00502-4

[B16] Manickan E Karem KL Rouse BT DNA vaccines–a modern gimmick or a boon to vaccinology? Crit Rev Immunol 1997 17 139 154 10.1615/CritRevImmunol.v17.i2.20 9094450

[B17] Whalen RG Davis HL Use of plasmid DNA for direct gene transfer and immunization Ann N Y Acad Sci 1995 772 21 29 10.1111/j.1749-6632.1995.tb44728.x 8546395

[B18] Mansouri S Lavigne P Corsi K Benderdour M Beaumont E Fernandes JC Chitosan-DNA nanoparticles as non-viral vectors in gene therapy: strategies to improve transfection efficacy Eur J Pharm Biopharm 2004 57 1 8 10.1016/S0939-6411(03)00155-3 14729076

[B19] Islam MA Firdous J Choi YJ Yun CH Cho CS Design and application of chitosan microspheres as oral and nasal vaccine carriers: an updated review Int J Nanomedicine 2012 7 6077 6093 23271909 10.2147/IJN.S38330 PMC3526152

[B20] Oda K Matsuda H Murakami T Katayama S Ohgitani T Yoshikawa M Adjuvant and haemolytic activities of 47 saponins derived from medicinal and food plants Biol Chem 2000 381 67 74 10722052 10.1515/BC.2000.009

[B21] Adams MM Damani P Perl NR Won A Hong F Livingston PO Ragupathi G Gin DY Design and synthesis of potent Quillaja saponin vaccine adjuvants J Am Chem Soc 2010 132 1939 1945 10.1021/ja9082842 20088518 PMC2820154

[B22] Dewey C Charbonneau G Carman S Hamel A Nayar G Friendship R Eernisse K Swenson S Lelystad-like strain of porcine reproductive and respiratory syndrome virus (PRRSV) identied in Canadian swine Can Vet J 2000 41 493 494 10857036 PMC1476209

[B23] Kim SH Roh IS Choi EJ Lee C Lee CH Lee KH Lee KK Song YK Lee OS Park CK A molecular analysis of European porcine reproductive and respiratory syndrome virus isolated in South Korea Vet Microbiol 2010 143 394 400 10.1016/j.vetmic.2009.11.039 20053505

[B24] Amonsin A Kedkovid R Puranaveja S Wongyanin P Suradhat S Thanawongnuwech R Comparative analysis of complete nucleotide sequence of porcine reproductive and respiratory syndrome virus (PRRSV) isolates in Thailand (US and EU genotypes) J Virol 2009 16 6 143 10.1186/1743-422X-6-143 PMC2753317 19754975

[B25] Zhuang JS Yuan SS Zhang JW Construction and analysis of full length genomic cDNA clone for European-type PRRSV low virulent strain Chinese Veterinary Science 2008 38 08 658 664

[B26] Huang MQ Zheng M Chen SL Che YL Jiang B Wang LB Wei H Zhou LJ Zhuang XS Chen SY Differences in the physicochemical characteristics and pathogenicity between European type PRRSV and North Ametican type PRRSV Fujian Journal of Agricultural Science 2012 27 1 24 26

[B27] Chen NH Cao Z Yu XL Deng X Zhao T Wang L Liu Q Li X Tian K Emergence of novel European genotype porcine reproductive and respiratory syndrome virus in mainland China J Gen Viro 2011 92 880 892 10.1099/vir.0.027995-0 21216986

[B28] Zhao P Tai C Cui ZZ Evolution of porcine reproductive and respiratory syndrome virus under antibody immune selective pressures Sci China Life Sci 2010 40 10 952 962 10.1007/s11427-012-4364-1 23015127

[B29] Zhao P Ma CT Dong X Cui Z Evolution of quasispecies diversity for porcine reproductive and respiratory syndrome virus under antibody selective pressures Sci China Life Sci 2012 42 8 662 667 10.1007/s11427-012-4364-1 23015127

[B30] Bernkop-Schnurch A Chitosan and its derivatives: potential excipients for peroral peptide delivery systems Int J Pharm 2000 194 1 13 10.1016/S0378-5173(99)00365-8 10601680

[B31] Porporatto C Bianco ID Correa SG Local and systemic activity of the polysaccharide chitosan at lymphoid tissues after oral administration J Leukoc Biol 2005 78 62 69 10.1189/jlb.0904541 15809287

[B32] Illum L Jabbal-Gill I Hinchcliffe M Fisher AN Davis SS Chitosan as a novel nasal delivery system for vaccines Adv Drug Deliv Rev 2001 51 81 96 10.1016/S0169-409X(01)00171-5 11516781

[B33] Guang Liu W De Yao K Chitosan and its derivatives–a promising non-viral vector for gene transfection J Control Release 2002 83 1 11 10.1016/S0168-3659(02)00144-X 12220833

[B34] Mao HQ Roy K Troung-Le VL Janes KA Lin KY Wang Y August JT Leong KW Chitosan-DNA nanoparticles as gene carriers: synthesis, characterization and transfection efficiency J Control Release 2001 70 399 421 10.1016/S0168-3659(00)00361-8 11182210

[B35] Xu Q Wang CH Pack DW Polymeric carriers for gene delivery: chitosan and poly(amidoamine) dendrimers Curr Pharm Des 2010 16 2350 2368 10.2174/138161210791920469 20618156 PMC3471158

[B36] Kensil CR Saponins as vaccine adjuvants Crit Rev Ther Drug Carrier Syst 1996 13 1–2 1 55 8853958

[B37] Kumar M Behera AK Matsuse H Lockey RF Mohapatra SS Intranasal IFN-γ gene transfer protects BALB/c mice against respiratory syncytial virus infection Vaccine 1999 18 5–6 558 567 10519947 10.1016/s0264-410x(99)00185-1

[B38] Kim JJ Maguire HC Jr Nottingham LK Morrison LD Tsai A Sin JI Chalian AA Weiner DB Coadministration of IL-12 or IL-10 expression cassettes drives immune responses toward a Th1 phenotype J Interferon Cytokine Res 1998 18 7 537 547 10.1089/jir.1998.18.537 9712370

[B39] Martínez-Lobo FJ Díez-Fuertes F Simarro I Castro JM Prieto C Porcine reproductive and respiratory syndrome virus isolates differ in their susceptibility to neutralization Vaccine 2011 29 6928 6940 21807060 10.1016/j.vaccine.2011.07.076

[B40] Lopez OJ Osorio FA Role of neutralizing antibodies in PRRSV protective immunity Vet Immunol Immunopathol 2004 102 3 155 163 10.1016/j.vetimm.2004.09.005 15507302

[B41] Martelli P Gozio S Ferrari L Rosina S De Angelis E Quintavalla C Bottarelli E Borghetti P Efficacy of a modified live porcine reproductive and respiratory syndrome virus (PRRSV) vaccine in pigs naturally exposed to a heterologous European (Italian cluster) field strain: clinical protection and cell-mediated immunity Vaccine 2009 27 3788 3799 10.1016/j.vaccine.2009.03.028 19442420

[B42] Lowe JE Husmann R Firkins LD Zuckermann FA Goldberg TL Correlation of cell-mediated immunity against porcine reproductive and respiratory syndrome virus with protection against reproductive failure in sows during outbreaks of porcine reproductive and respiratory syndrome in commercial herds J Am Vet Med Assoc 2005 226 1707 1711 10.2460/javma.2005.226.1707 15906573

[B43] Meier WA Galeota J Osorio FA Husmann RJ Schnitzlein WM Zuckermann FA Gradual development of the interferon-gamma response of swine to porcine reproductive and respiratory syndrome virus infection or vaccination Virology 2003 309 18 31 10.1016/S0042-6822(03)00009-6 12726723

[B44] Bassaganya-Riera J Thacker BJ Yu S Strait E Wannemuehler MJ Thacker EL Impact of immunizations with porcine reproductive and respiratory syndrome virus on lymphoproliferative recall responses of CD8+ T cells Viral Immunol 2004 17 25 37 10.1089/088282404322875430 15018660

[B45] Parida R Cell-Mediated Immunity in Porcine Reproductive and Respiratory Syndrome Virus Master thesis 2012 University of Nebraska-Lincoln, Veterinary and Biomedical Sciences Department

[B46] Gimeno M Darwich L Diaz I de la Torre E Pujols J Martín M Inumaru S Cano E Domingo M Montoya M Mateu E Cytokine profiles and phenotype regulation of antigen presenting cells by genotype-I porcine reproductive and respiratory syndrome virus isolates Vet Res 2011 42 9 10.1186/1297-9716-42-9 21314968 PMC3037899

[B47] Zhou Z Liu Q Hu D Yue X Yu X Zhang Q Gu X Ni J Li X Zhai X Tian K Complete genome sequence of a European genotype porcine reproductive and respiratory syndrome virus in china Genome Announc 2013 1 3 175 13 10.1128/genomeA.00175-13 PMC3650434 23661475

[B48] Reed LJ Muench H A simple method of estimating fifty percent end points Am J Hyg 1938 27 493 497

[B49] Shen G Jin N Ma M Jin K Zheng M Zhuang T Lu H Zhu G Jin H Jin M Huo X Qin X Yin R Li C Li H Li Y Han Z Chen Y Jin M Immune responses of pigs inoculated with a recombinant fowlpox virus coexpressing GP5/GP3 of porcine reproductive and respiratory syndrome virus and swine IL-18 Vaccine 2007 25 4193 4202 10.1016/j.vaccine.2007.03.010 17418456

[B50] Han S Jin NY Lu HJ Jin KS Nan WL Mu WF Zhao CQ Immune responses of mice inoculated with a recombinant fowlpox virus expressing ORF5/ORF6 of porcine reproductive and respiratory syndrome virus Chin J Vet Sci 2010 30 1 1 5

